# Supplementary material for: Content-rich biological network constructed by mining PubMed abstracts
Source: BMC Bioinformatics. 2004 Oct 8;5:147. doi: 10.1186/1471-2105-5-147 (PMC528731; doi:10.1186/1471-2105-5-147)
Supplement: Additional File 5 — The original Chilibot query results of the term "long-term potentiation (LTP)" and 22 other terms, limiting the latest references analyzed to the years 1990, 1995, 2000, and 2004. [file 1471-2105-5-147-S5.bz2 › chilibotAdditionalFile5/ltp2004/html/SYNAPSIN I_ARC.html]

 


 **SYNAPSIN I** and **ARC** 
  
Found 1 abstracts in PubMed,  **1 abstracts were retrieved and analyzed**.  


---

 Search Google  |
 PDF files only 
|  EDU domain only 

---

- Brain Res Mol Brain Res, 2002   **Plasticity driven gene expression in the rat retina.** .
  Animals exposed to an enriched environment display features of neural plasticity such as an increased brain volume, enhanced number of dendritic spines, as well as enlarged synapses.
  Here we report the first description of molecular plasticity in the mammalian retina, as revealed by gene expression.
  A marked upregulation of both NGFI A and **Arc**, two candidate plasticity genes, was observed in adult rats that had been exposed to an enriched environment for 3 weeks.
  This increase was paralleled by an increase in the expression of the late genes GAP 43 and **Synapsin I**, which also indicated changes in retinal connectivity.
  Our results suggest that both NGFI A and **Arc** may regulate mechanisms of plasticity that had been invoked by heightened complexity of the visual environment.
